# Links among inflammation, sexual activity and ovulation: Evolutionary trade-offs and clinical implications

**DOI:** 10.1093/emph/eov029

**Published:** 2015-12-16

**Authors:** Tierney K. Lorenz, Carol M. Worthman, Virginia J. Vitzthum

**Affiliations:** ^1^The Kinsey Institute, Indiana University, Morrison Hall 313, 1165 E 3rd Street, Bloomington, IN 47405, USA;; ^2^The Center for Integrative Study of Animal Behavior, Indiana University, Bloomington, IN 47405, USA;; ^3^Laboratory for Comparative Human Biology, Department of Anthropology, Emory University, 214 Anthropology, 1557 Dickey Drive, Atlanta, GA 30322, USA;; ^4^Evolutionary Anthropology Laboratory, Department of Anthropology, Indiana University, Student Building 130, 701 E. Kirkwood Avenue, Bloomington, IN 47405, USA

## Abstract

**Background and objectives:** We examined a mechanism that may coordinate trade-offs between reproduction and immune response in healthy women, namely, changes in inflammation across the ovarian cycle.

**Methodology:** We investigated C-reactive protein (CRP), an inflammation marker, across two consecutive ovarian cycles in 61 Bolivian women. Participants provided saliva samples every other day, and dried blood spots on 5–6 days spread across weeks 2–3 of each cycle. Cycles were characterized as ovulatory/anovulatory based on profiles of reproductive hormones. Participants also reported whether they were sexually partnered with a male or sexually abstinent during the study.

**Results:** High early-cycle, but not late-cycle, CRP was associated with anovulation. High inflammation at the end of one cycle was not associated with anovulation in the subsequent cycle. Among ovulatory cycles, women with sexual partners had significantly lower CRP at midcycle, and higher CRP at follicular and luteal phases; in contrast, sexually abstinent women had little cycle-related change in CRP. In anovulatory cycles, partnership had no effect on CRP. CRP varied significantly with socioeconomic status (higher in better-off than in poorer women).

**Conclusions and implications:** These findings suggest that the cycle-specific effect of inflammation on ovarian function may be a flexible, adaptive mechanism for managing trade-offs between reproduction and immunity. Sociosexual behavior may moderate changes in inflammation across the ovarian cycle, suggesting that these shifts represent evolved mechanisms to manage the trade-offs between reproduction and immunity. Clinically, these findings support considering both menstrual cycle phase and sexual activity in evaluations of pre-menopausal women’s CRP concentrations.

## Abstract (precis)

Inflammation in healthy sexually active women decreased at midcycle, around ovulation, which may have evolved to promote conception.

## BACKGROUND AND OBJECTIVES

Inflammation increasingly has been regarded as a health risk, in part due to its association with a variety of chronic conditions including metabolic syndrome, pain disorders, cardiovascular disease (CVD) and depression [1–6]. Such evidence has tended to obscure the long-recognized adaptive function of inflammation to signal physical challenges (infection or damage) and coordinate immune response with other physiologic functions including reproduction [7–13].

Evolutionary theory argues that because the immune system and reproduction each require substantial resources (e.g. energy, micronutrients), an organism must typically trade off finite resource allocations between these demands; that is, concurrent maximum investments in reproduction and immune defenses are unlikely [11, 14–16] with some possible exceptions such as following acute exposure to cortisol [17]. In addition to competition for resources, the inherent non-specificity of inflammation makes it a potent immune response to innumerable foreign cells including, potentially, sperm or a conceptus. Thus, natural selection may favor a transient dampening of maternal immune defense to bodily threats so as to increase the chances for successful reproduction. This dampening, however, may come at significant health costs to women (e.g. infertility, autoimmune diseases, sexually transmitted infections) [18–23].

Such trade-offs have long been studied both theoretically and empirically. In *On the Origin of Species*, Darwin wrote (citing Goethe), ‘In order to spend on one side, nature is forced to economize on the other side’ [24]. This simple calculus is at the heart of varied allocation compromises (for assessments of immune-reproduction trade-offs in humans, see e.g. McDade [25], Muehlenbein and Bribiescas [26], Abrams and Miller [13] and Clancy [9]; a few examples from the vast literature on non-human animals are Lochmiller and Deerenberg [10], Sheldon and Verhulst [12], Norris and Evans [27] and Demas et al. [28]).

We tested three hypotheses grounded in evolutionary theory regarding the trade-offs between inflammation, sexual activity and ovulation in a sample of healthy Bolivian women not using hormonal contraception. To track changes in inflammation during the ovarian cycle, we measured C-reactive protein (CRP), an acute-phase protein produced by the liver in response to signals from immune agents such as macrophages [29]. Low circulating concentrations of CRP reflect a broad array of processes related to ongoing baseline somatic maintenance, but rise abruptly within about 2 h of an acute insult. Given its relatively short half-life (19 h), CRP concentrations reflect the rate of synthesis, driven directly by innate inflammatory processes [30]. These features make CRP a particularly valuable biomarker of inflammation and, due to its increasingly common use in clinical settings to predict risk of conditions such as CVD [31–33], its use in our study affords the opportunity to evaluate the clinical significance of our findings.

**Hypothesis 1:** *High CRP during the follicular phase is associated with an increased risk of anovulation.* We predict that women experiencing high inflammation (as indexed by high CRP) will be less likely to ovulate, instead favoring physiologic processes related to inflammation such as defense or healing. Anovulation is an unambiguous signal of terminated effort in the current reproductive opportunity. Such temporary suspension of reproductive investment may be evolutionarily adaptive [34–40]. As well as diverting the resources necessary to mount an adequate immune response (including inflammation), a conception during periods of high inflammation might have a substantially reduced chance of developing into a viable offspring because the embryo may be harmed by the activated maternal immune system, diversion of energetic resources to the immune system and, potentially, the presence of pathogens [41, 42]. Natural selection would, therefore, favor not conceiving in a cycle in which heightened immune defenses are needed.

**Hypothesis 2:** *The effect of high inflammation on ovulation is cycle-specific.* Because natural selection over a lifetime favors reproduction, acute inflammation generally may be expected to have short-term rather than prolonged effects on ovarian functioning in adulthood. Specifically, we predict that ovarian activity in each cycle responds independently to transient inflammation, that is, inflammation during one cycle would not predict anovulation in the next.

Such acute responsivity would allow the body to opt for anovulation flexibly by reevaluating and responding to immune status at each cycle. Evidence suggests that the ovarian system uses short-term cycle-specific strategies (as well as other strategies, e.g. across sequential cycles [43]) to coordinate with other physiological systems (e.g. under stressful conditions, cross-talk between the HPA- and HPG-axes coordinates suppression of reproductive investment [44, 45]). Similarly, the endocrine and immune systems engage in cross-talk during pregnancy to evaluate physical and psychosocial stressors to determine ongoing investment in the pregnancy versus premature delivery [46–48].

**Hypothesis 3:** *Patterns of inflammation are moderated by sexual partnership status such that, relative to unpartnered women, sexually active women will have higher inflammation overall, but exhibit a decrease in inflammation corresponding to the occurrence and timing of ovulation.* Sexually active women are at a greater risk than abstinent women for sexually transmitted infections and/or genital irritation. Therefore, we predict that sexually partnered women will exhibit relatively higher CRP concentrations during those portions of the cycle when the probability of conception is lower (i.e. the early to mid-follicular and mid- to late luteal phases). We also predict that in healthy sexually active females, the immune system will temporarily down-regulate non-specific defenses (such as inflammation) at ovulation to mitigate disruption of conception. Indeed, two very recent US-based studies have found that some immune markers differ significantly in sexually active versus abstinent women [49–51]. However, to date there are few data regarding the interaction of sexual activity, cycle ovulation status and inflammation in healthy premenopausal women. This is a significant gap in our knowledge, as there is reason to expect that sexual activity may have different effects on immune response at fecund points of ovulatory cycles (i.e. around ovulation) than during other times, or in comparison to anovulatory cycles.

We selected this Bolivian population for testing these three hypotheses because it affords an opportunity to understand both cycle-associated variation in CRP and reproductive-immune trade-offs in women living in very demanding conditions [52, 53]. The overwhelming bulk of research on CRP has been conducted in US and European populations. To complement this work, several researchers have called for studies in a broader range of living conditions, health care practices and pathogen burdens [8, 25, 54–58]. Dissimilar habitats, particularly when experienced early in life, are thought to differentially influence the trajectory of immune responses and the associated physiological trade-offs [8, 25, 54]. Groundbreaking work on variation in CRP in both adults and children has been conducted in a handful of non-Western (i.e. neither European or Euro-American [59]) populations in Siberia [60, 61], Ecuadorian [62, 63] and Bolivian [57, 64–67] Amazonia, and the Philippines [68–72]. These studies addressed questions regarding the prevalence of chronic inflammation, risks for cardiovascular and other chronic diseases (particularly under conditions of endemic infectious disease), aging, depression, links between early life conditions and later life health, and trade-offs between immune functioning and growth.

Despite these advances, however, variation in immune biomarkers during the ovarian cycle, or in relation to women’s reproductive functioning more generally, has been relatively unexamined in non-Western populations. One exception is a study from the Philippines that reported significantly higher CRP in late gestation compared with nulliparous women but no difference in CRP with respect to breastfeeding status [56]. The authors suggested that during human evolutionary history, adult females may have spent more time in a pro-inflammatory state compared with other great apes experiencing fewer pregnancies and longer lactation durations, but they also cautioned that the health or energetic costs of such a pro-inflammatory shift are uncertain.

Studies on whether CRP varies in concert with cyclical changes in one or another reproductive hormone have been conducted in Austria [73], Switzerland [74, 75], Italy [76], Poland [55] and the USA [77, 78]. For the most part, this work has evaluated proximate mechanisms (in particular, the hypothesized pro- or anti-inflammatory effects of endogenous reproductive hormones) that may cause serum CRP to rise or fall. In the one study to address these questions using a urinary CRP biomarker, Clancy *et al.* [55] adopted an evolutionary approach, predicting that higher CRP would be correlated with lower progesterone and estradiol in a sample of rural Polish women. Most of these studies also considered whether serum CRP measurements should be adjusted for menstrual cycle phase.

Collectively, the results from these studies (considered further in the Conclusions and Implications section) are quite mixed (and even contradictory for a given sample), perhaps due to differences in study design and analytical approaches (e.g. inattention to cycle ovulation status). In particular, the focus on absolute concentrations of reproductive steroids may need to be reconsidered because these hormones are highly variable and because absolute reproductive hormone concentrations are an ambiguous signal of reproductive investment.

There is now substantial evidence attesting to the considerable natural variation across cycles (within an individual), women (within a population) and populations in concentrations of ovarian steroids during normal spontaneous menstrual cycles [38, 79]. It has also been demonstrated that women with progesterone concentrations lower than some population average, and populations with such concentrations lower than those observed in US women, are not necessarily subfecund [80, 81]. In the face of such variability, one may fail to find a correlation between CRP and some steroid concentration if the typically small samples have insufficient statistical power (notably, several of the studies on CRP–hormone associations specifically reported a concern with sample size). Even given large sample sizes, CRP–hormone associations may vary across women because of individual differences in the interactions of baseline CRP and hormone concentrations (a subject for much needed research).

A key implication of such marked hormonal variability for studies of evolved immunity-reproduction trade-offs is that lower steroid hormone concentrations need not necessarily indicate a reduction in reproductive effort. Although ecological stressors may be accompanied by a reduction in ovarian steroids that suggest reduced (but still not zero) investment in reproduction, it does not follow that normal inter-cycle variability in hormone concentrations is necessarily a reflection of varying reproductive effort. In contrast, anovulation is an unambiguous signal of terminated effort in the current reproductive opportunity. For this reason, we have chosen to evaluate hypothesized immune-reproduction trade-offs using the ovulatory status of a cycle as the marker of reproductive investment.

Sexual partnership is likely to be a key factor moderating immune-reproduction trade-offs in women that has yet to be considered in studies of cycle-associated CRP variation. Beginning with Metcalf’s ground breaking work on ovulation rates in New Zealand women, several studies have shown that sexual partnership is associated with higher ovulation rates, particularly when women are living apart from close relatives [82–85]. Furthermore, very recent work has demonstrated that some immune parameters differ significantly in sexually active versus abstinent US women [49–51]. These findings all point to a significant role for the social and sexual environments as predictors of ovarian and immune functioning.

As previously noted, one can reasonably expect natural selection to favor a transient dampening of maternal immune defenses so as to increase the chances for successful reproduction. In addition, one can also reasonably expect that natural selection would differentially favor such dampening, including the accompanying increased health risks, in those women who are actually at risk for conception, i.e. in sexually active women as opposed to sexually abstinent women.

Although we draw widely on a preceding body of evolutionary theory and evidence, to the best of our knowledge, the specific hypotheses we propose to test have not been previously evaluated. In addition, our study of hypothesized immune-reproduction trade-offs in a non-Western population with higher pathogen load than is typical of wealthier countries is itself a test of whether such cycle-associated variation in inflammation is likely to be an evolved and adaptive mechanism or a newer phenomenon reflective of some aspect of modern life.

### Clinical implications

In the concluding section, we discuss the significance of our findings for clinical practice. Because serum CRP is a commonly used biomarker for assessing the risk of CVD, and both some prior work and the hypotheses we tested suggest that CRP is likely to vary over the ovarian cycle, we evaluate the consequences of such changes for assessing CVD risk in nominally healthy adult women. In a recent study of US women, Schisterman *et al.* [86] found that, due to natural variation in CRP across the menstrual cycle, the same women were twice as likely to be diagnosed as high CVD risk during menses than at any other point in the cycle, indicating that failure to account for timing within the ovarian cycle likely leads to clinical misinterpretation of inflammation biomarkers in premenopausal women.

## METHODS

The data presented here were collected as part of a study of the determinants and consequences of variation in reproductive functioning in urban-dwelling Bolivian women [52, 87]. Participants were recruited and samples were collected from May through mid-August 1995. All samples were treated identically during sampling, storage, shipping and analysis (details presented further below). Saliva assays were performed during 1996, and assays of dried blood spots (DBS) assays were performed during November 2004 through February 2005.

### Participants

Sixty-one women (age 23–35 years, mean = 28.13 years) were recruited from La Paz, the capital of Bolivia, and Pasenkeri, a poor community on the outskirts of La Paz. Participants were recruited via announcements (to meetings at the Pasenkeri community center and to classes at the university) and word of mouth. Inclusion criteria comprised having regular ovarian cycles between 25 and 35 days inclusive, stable weight (no more than ±2 kg in the last 6 months) and continuous residence at high altitude (>3500 m) since early childhood. Exclusion criteria included use of prescription medications (including hormonal contraceptives) during the previous 6 months, pregnancy or lactation during the previous 6 months, or any known current or previous sexually transmitted infection or reproductive disorder. Screening interviews and informed consent procedures were conducted in the participant’s native language (Spanish or Aymara). All procedures were approved by the Institutional Review Board at the University of California at Riverside.

### Saliva and blood spot collections

For two complete consecutive cycles, serial 5 ml saliva samples were collected every other day (either Monday–Wednesday–Friday or Tuesday–Thursday–Saturday; the same weekly schedule was maintained throughout the study for a given participant) according to an established protocol [88]. During the second and third week of each cycle, beginning in the mid-follicular phase (i.e. beginning on cycle Day 6 or 7 or 8) and continuing through the presumed mid-luteal phase (i.e. depending on the start day, ending on cycle Day 21 or 22 or 23 or 24), blood spot samples were collected concurrently with saliva samples on 5–6 occasions. Per our published procedure, after collection, blood spot sample papers were dried for 3–4 h at ambient temperature, then placed in a sealed bag with a desiccant packet and stored in a 2°C laboratory refrigerator until transported with cold packs, within 6 weeks of collection, to Emory University for processing [89]. Upon receipt, DBS were stored frozen at −28°C until assayed. All saliva samples were treated with sodium azide (an antimicrobial preservative) and stored at cool ambient temperature (averaging ∼15°C) until shipped to the USA, where they were stored frozen at −28°C until assayed [52].

### CRP assay

DBS were assayed for CRP using a high-sensitivity europium-labeled biotin–strepavidin system that improved on a previously published method [90] (additional details are provided in Supplementary Data, permanently archived at http://hdl.handle.net/2022/20415 29 November 2015, date last accessed). Assay minimum detectable dose was 0.010 mg/l; coefficients of variation (CV) were low: intra-assay (1.2–2.0%) and inter-assay (10.9–14.9%; see archived Supplementary Data for all values). As reported elsewhere, a validation study using matched serum and DBS samples was performed and found a close linear correlation such that serum equivalents can be computed from DBS values using this algorithm: high-sensitivity CRP mg/l = 1.38 * (DBS CRP mg/l) − 0.97 [91].

A total of 639 DBS from 61 women collected across two cycles were assayed for CRP, of which 65 measurements were excluded from further analyses (9 from one participant who conceived during her first cycle; and 56 from 10 cycles across 10 women that could not be characterized as either ovulatory or anovulatory (see section below on ascribing ovulation)). Of the remaining 574 measurements, 23 were >4 mg/l (indicating acute inflammatory response). These values were retained; however, due to these few very high values, the raw CRP data were significantly right-skewed. Therefore, we used the natural log of CRP in all analyses. It should be noted that in no participant was CRP >4 mg/l for longer than three consecutive samples (at most, 6 days total over two cycles); that is, observed CRP elevations were acute, not chronic. Summary characteristics of CRP are presented in Table 1. Note that average CRP values are taken across repeated measures which are not statistically independent; thus, the *F* and *P* values for CRP contrasts in Table 1 are presented for general interpretation only.

**Table 1.** Descriptive statistics for study sample

	Partnered women (*N* = 31)				Unpartnered women (*N* = 29)				Total (*N* = 60)		Contrast partnered versus unpartnered women		Contrast ovulatory versus anovulatory cycles	
	Ovulatory cycle		Anovulatory cycle		Ovulatory cycle		Anovulatory cycle							
	*M*	SD	*M*	SD	*M*	SD	*M*	SD	*M*	SD	*F*	*P*	*F*	*P*
Age	31.00	3.23	26.84	3.19	26.79	3.38	27.13	4.21	28.19	3.95	6.60	0.01		
BMI	24.33	3.47	25.39	2.68	22.86	2.73	25.79	2.47	24.18	3.16	1.88	0.18		
Cycle length	26.97	3.05	27.05	1.86	28.26	2.84	28.47	4.93	27.66	3.22	4.60^a^	0.03^a^	0.00	0.98
Forward cycle day of ovulation	15.09	2.89			16.03	2.77			15.59	2.88	2.02^a^	0.16^a^		
CRP (mg/l), average across cycle	0.82	1.48	0.92	1.18	0.72	1.24	1.17	1.77	0.85	1.40	0.00^a^	0.99^a^	4.56^a^	0.03^a^

a Note that repeated measures are not statistically independent, therefore these F and P values are not useful for statistical inference.

### Hormone assays and ascribing ovulation

DBS for one of a woman’s two cycles (selected arbitrarily, without regard to CRP concentrations or any other cycle characteristics) were assayed for progesterone (P4), estradiol (E2), luteinizing hormone (LH) and follicle stimulating hormone (FSH) using previously published methods [89]. CVs were acceptable (intra-assay range, 2.5–19.8%, inter-assay range, 6.1–34.8%; assay performance details are in Supplementary Data, archived at http://hdl.handle.net/2022/20415). Saliva samples were assayed for unbound (free) P4, the results of which have been previously published [52].

We used multiple hormonal criteria to ascribe ovulation to each cycle. A cycle was characterized as ‘ovulatory’ if mean-peak-salivary-P4 >110 pmol/l [52]. For cycles in which DBS were assayed for hormones, assessment of ovulatory status was supplemented by the following criteria: a clear mid-cycle surge of LH and FSH, an accompanying relative peak of E2 at mid-cycle and/or a late-cycle rise in serum P4. Seventy-three cycles were characterized as ovulatory (Cycle 1, *N *= 36; Cycle 2, *N* = 37). Cycles were characterized as ‘anomalous’ and dropped from further analyses (Cycle 1, *N* = 7; Cycle 2, *N* = 3) if the salivary and serum P4 criteria were not in agreement, or if unusual features were present (e.g. an apparent late-cycle peak in LH). Cycles lacking any manifest features of ovulation were characterized as ‘anovulatory’ (Cycle 1, *N *= 17; Cycle 2, *N* = 17). Ascribed ovulation status was independent of whether or not DBS hormonal data were used (Fisher’s Exact Test, *P *= 0.45). For cycles categorized as ovulatory, the day of ovulation was determined from the LH/FSH peak and/or the post-ovulatory rise in salivary P4.

### Other measures

At the initial interview, participants reported their age and current sexual partnership status (with [*n* = 31] or without [*n* = 29] a male partner). Given the patterns of contraception use and pregnancy within this sample and within this culture, we can conclude that the partnered women were regularly sexually active [52]. Unpartnered women reported that they did not engage in sexual activity during the study period (and based on the hormonal data, no conceptions occurred during the study period in this subsample); thus, consistent with this culture’s mores, it is likely that unpartnered women were rarely, if ever, sexually active. Of note, ovulation was not associated with partnership status in this sample (*χ^2^*(1) = 0.80,*P* = 0.41). Participants were also measured for height and weight; from this, body mass index (BMI) was calculated as weight/height^2^. Partnered and unpartnered women did not differ in the protocols followed, the number of samples collected per participant, or in the collection, handling and assaying of their samples.

### Statistical analyses

The archived data used in the following analyses are freely and permanently available at http://hdl.handle.net/2022/20420. All statistics were conducted with SPSS version 22. Because CRP is positively correlated with age and BMI in at least some populations [92], all our fitted models included these potential confounders.

To test our first hypothesis (CRP is higher during the follicular phase in anovulatory cycles), we fitted a repeated measures mixed model (outcome variable was lnCRP) with random intercepts by participant (to adjust for individual differences in baseline CRP), controlling for age (centered at 28, i.e. near mean age), BMI (centered at 24, i.e. near mean BMI) and socioeconomic status (SES, dichotomized as poorer or better-off, see Ref. [52] for details). As the dependent variable (lnCRP) was continuous, we assumed a Gaussian distribution. Because anovulatory cycles cannot be evaluated with respect to the day of ovulation, the repeated measures variables were cycle and reverse day (i.e. day relative to first day of subsequent cycle). We defined a phase variable to allow for possible differences by cycle phase, coded as 0 if reverse day < −14 (i.e. early cycle (approximates follicular phase)) or 1 if reverse day ≥−14 (i.e. late cycle (approximates luteal phase in ovulatory cycles)). We parameterized time dependence with two terms: the interaction of phase with ovulation status (ovulatory/anovulatory) and the interaction of phase with ovulation status with (reverse day + 14)^2^. We used an autoregressive model of the repeated measures covariance; this model type assumes that the best predictors of each repeated measure are the measures closest to it in time (e.g. the best predictors of CRP at Day −10 are the measures at Day −8 and Day −12).

To test our second hypothesis (the effect of high inflammation on ovulation is cycle-specific), we coded a participant’s first cycle as exhibiting high inflammation late in the cycle if there was a high CRP value in Day 14 or later. We tested two potential cutoffs: untransformed CRP >2.02 mg/l, corresponding to the mean (*M* = 0.72 mg/l), plus one standard deviation (SD = 1.31 mg/l), across all Cycle 1 luteal phase samples; and untransformed CRP >3.0 mg/l, the cutoff most often used in Western populations (as recommended by the American Heart Association (AHA) [32]). Within Cycle 1, 15% of the cycles had samples over the >2.02 mg/l cutoff and characterized as ‘high late-cycle inflammation’, and 5% of the cycles had at least one sample that met criteria under the >3.0 mg/l cutoff. To examine whether anovulation in the subsequent cycle was independent of late-cycle high inflammation in the first cycle, we conducted Fisher’s Exact Test for independence.

To test our third hypothesis (patterns of inflammation are moderated by sexual partnership status), we fitted a pair of repeated measures mixed models, each with lnCRP as the outcome variable and random intercepts by participant (similar to our test of Hypothesis 1). We controlled for centered age, centered BMI and SES. We used an autoregressive model of the repeated measures covariance and assumed a Gaussian distribution. For the model fitted to those data from ovulatory cycles, the repeated measures variables were cycle and day-relative-to-ovulation. In this model, phase was coded as 0 if day-relative-to-ovulation < 0 (i.e. follicular) or 1 if day-relative-to-ovulation ≥0 (i.e. luteal). We parameterized time dependence with two terms: the interaction of phase with partner status (partnered/no-partner) and the interaction of phase with partner status with (day-relative-to-ovulation)^2^. For the model fitted to those data from anovulatory cycles, phase was coded relative to reverse day (as in the test of Hypothesis 1) and the repeated measures variables were cycle and reverse day (also as in the test of Hypothesis 1). We parameterized time dependence with two terms: the interaction of phase with partner status and the interaction of phase with partner status with (reverse day + 14)^2^.

## RESULTS

### Hypothesis 1: High CRP during the follicular phase is associated with an increased risk of anovulation

**Figure 1.**
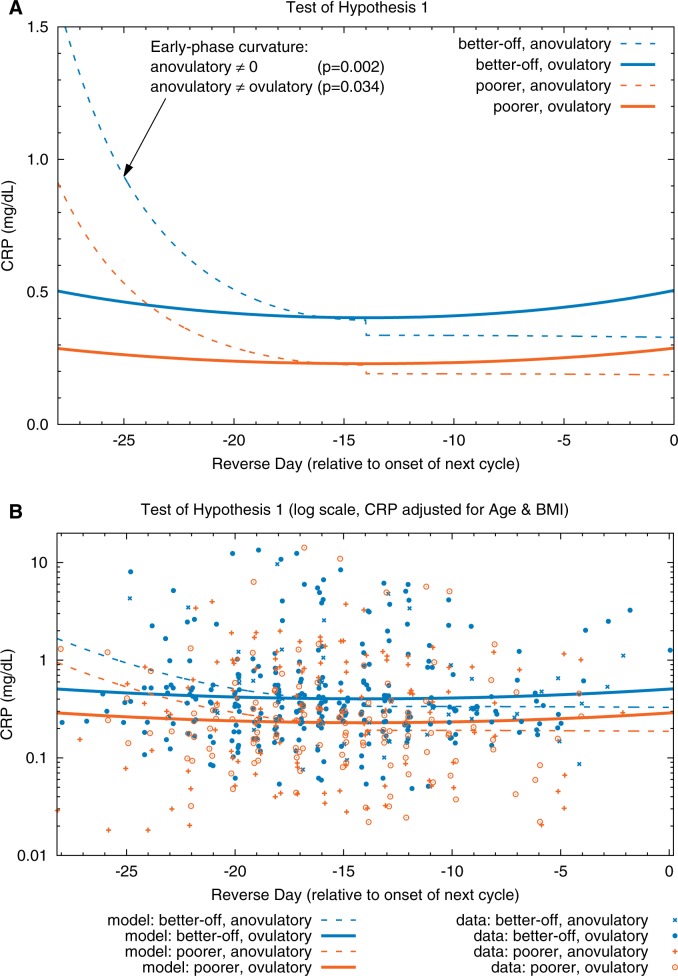
Test of Hypothesis 1. (**A**) Fitted models. (**B**) Fitted models and CRP data (adjusted for age and BMI) plotted on a log scale; adjusted CRP = exp(ln(observed CRP) − beta_age*(Centered_Age) − beta_BMI*(Centered_BMI)); data points are randomly dithered (slightly offset) on *x*-axis for ease of viewing. CRP in the early phase of anovulatory cycles (dashed curves) is significantly higher (*P* = 0.034) than in ovulatory cycles (solid curves). In anovulatory cycles, CRP is lower at mid-cycle than at the cycle’s beginning (fitted model curvature is significant at *P* = 0.002). In contrast to anovulatory cycles, CRP in ovulatory cycles is more stable over time (fitted model curvature is not significantly different from 0). CRP is significantly higher (*P*_SES_ = 0.034) in the cycles of better-off (blue curves) than in those of poorer women (orange curves)

Parameters for this model are given in Table 2 and plotted in Fig. 1. CRP concentrations in the early phase of anovulatory cycles (dashed lines) were significantly higher (*P *= 0.034) than those of ovulatory cycles (solid lines). In contrast, CRP did not differ between ovulatory and anovulatory cycles during late cycle. These findings support Hypothesis 1. An additional finding was that serum equivalent CRP concentrations were significantly higher (*P *= 0.034) in better-off women (blue lines) than in poorer women (orange lines).

**Table 2.** Hypothesis 1 model parameter estimates (outcome variable is lnCRP)

Fixed effects						
					95% CI for β	
Parameter	β	***SE***	***t***	*P*	Lower	Upper
Intercept	−1.474	0.221	−6.681	<0.001	−1.911	−1.036
Age^a^	0.022	0.031	0.732	0.467	−0.038	0.081
BMI^a^	0.197	0.041	4.850	<0.001	0.116	0.279
SES						
Better-off	0.563	0.260	2.169	0.034	0.044	1.083
Poorer	(reference)					
Ovulation * Phase						
Anovulatory, Early phase	−0.022	0.256	−0.087	0.931	−0.527	0.482
Anovulatory, Late phase	−0.179	0.271	0.663	0.508	−0.713	0.354
Ovulatory, Early phase	0.000	0.100	0.004	0.997	−0.196	0.197
Ovulatory, Late phase	(reference)					
Ovulation * Phase * (Reverse day + 14)^2^						
Anovulatory, Early phase	0.007	0.002	3.086	0.002	0.003	0.012
Anovulatory, Late phase	0.000	0.003	−0.038	0.970	−0.006	0.006
Ovulatory, Early phase	0.001	0.002	0.699	0.485	−0.002	0.004
Ovulatory, Late phase	0.001	0.002	0.489	0.625	−0.004	0.006
**Random effects**						
	**Variance**					
**Factor**	**Estimate**	***SE***				
Per-woman intercept	0.392	0.152				
**Repeated measures**						
	**Variance**					
**Factor**	**Estimate**	***SE***				
First-order autoregressive diagonal	0.998	0.124				
*ρ*	0.866	0.019				

a Centered.

### Hypothesis 2: The effect of inflammation on ovulation is cycle-specific

High inflammation in the latter half of a woman’s first cycle was not significantly associated with anovulation in the subsequent cycle, using either the sample-specific cutoff of CRP >2.02 mg/l (Fisher’s Exact Test, *P* = 0.47), or the Western-population cutoff of CRP >3.0 mg/l (Fisher’s Exact Test, *P* = 0.66). Thus, there was evidence for Hypothesis 2.

### Hypothesis 3: Patterns of inflammation are moderated by partnership status such that, relative to unpartnered women, sexually active women will have higher inflammation and a decrease in inflammation corresponding to the occurrence and timing of ovulation

**Figure 2.**
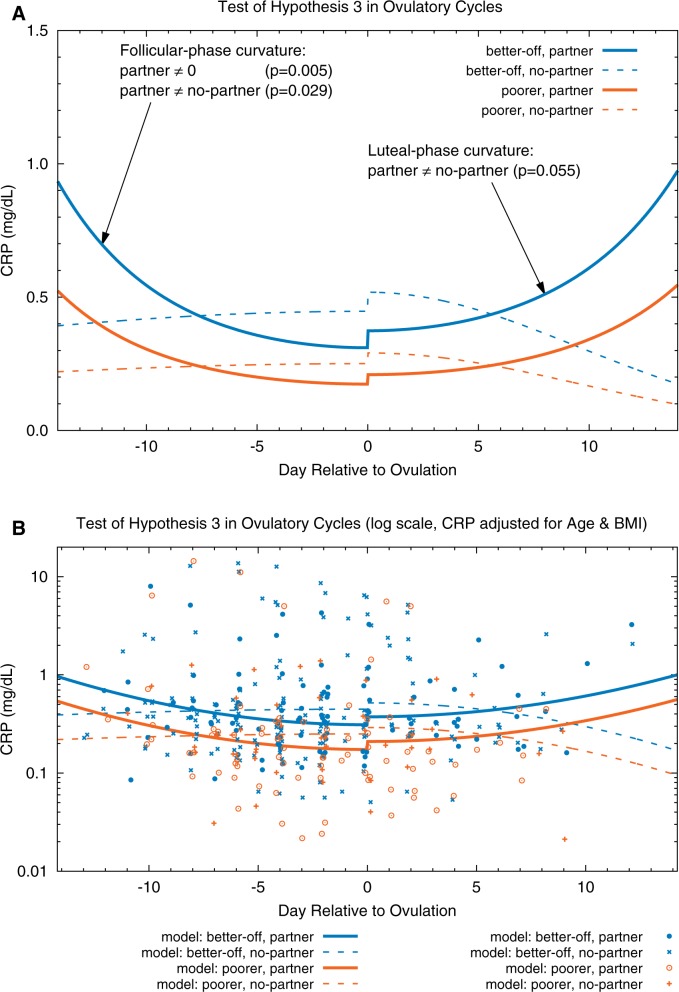
Test of Hypothesis 3 in ovulatory cycles. (**A**) Fitted models. (**B**) Fitted models and CRP data (adjusted for age and BMI) plotted on a log scale; adjusted CRP = exp(ln(observed CRP) − beta_age*(Centered_Age) − beta_BMI*(Centered_BMI)); data points are randomly dithered (slightly offset) on *x*-axis for ease of viewing. Interaction between partnership status, SES and cycle day predicted CRP in ovulatory cycles. CRP is significantly higher during the early follicular and late luteal phases (*P* = 0.029 and 0.055, respectively) in partnered (solid curves) than in unpartnered (dashed curves) women. In partnered cycles, CRP is lower around ovulation than at the cycle’s beginning (fitted model curvature is significant at *P* = 0.005). In contrast to partnered women, CRP in the ovulatory cycles of unpartnered women is more stable over time (fitted model curvature is not significantly different from 0). The small increases in CRP at ovulation are not statistically significant in these models

**Figure 3.**
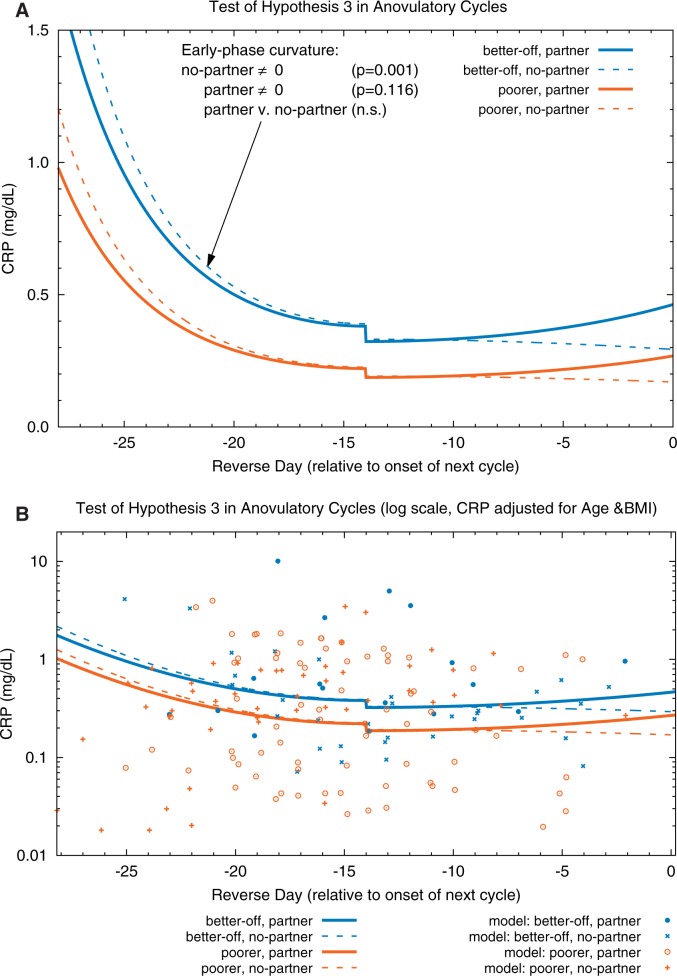
Test of Hypothesis 3 in anovulatory cycles. (**A**) Fitted models. (**B**) Fitted models and CRP data (adjusted for age and BMI) plotted on a log scale; adjusted CRP = exp(ln(observed CRP) − beta_age*(Centered_Age) − beta_BMI*(Centered_BMI)); data points are randomly dithered (slightly offset) on *x*-axis for ease of viewing. Partnership status does not significantly modify CRP in anovulatory cycles (i.e. within each SES [better-off = blue, poorer = orange], the solid [partnered] curves do not differ significantly from the corresponding dashed [unpartnered] curves). In unpartnered women, CRP is significantly higher during the beginning of the cycle than at mid-cycle (fitted model curvature is significant at *P* = 0.001); a similar cycle-day dependent change is seen in partnered women, but is not statistically significant (*P* = 0.116). The small decreases in CRP observed at mid-cycle are not statistically significant in these models

The parameters for these models are presented in Tables 3 and 4 and plotted in Figs 2 (ovulatory cycles) and 3 (anovulatory cycles). In ovulatory cycles, during the early follicular and late luteal phases, partnered women (solid curves) had significantly higher CRP concentrations (*P *= 0.029 and 0.055, respectively) than unpartnered women (dashed curves). (Although the curvatures of the same-color solid lines in the follicular and luteal phases are very similar, the respective statistical significances differ because there are fewer data points in the luteal phase.) Furthermore, CRP in partnered women was significantly lower during the peri-ovulatory period than during the early follicular phase (*P *= 0.005). In contrast, CRP in unpartnered women changed little throughout the cycle (i.e. the curvature of the model for unpartnered women was not significantly different from 0); in other words, there was no evidence of a peri-ovulatory decline in inflammation as was observed in the partnered women. In anovulatory cycles (Fig. 3), partnership status was not associated with any changes in CRP concentrations at any time during the cycle (i.e. the two blue curves do not differ significantly nor do the two orange curves). The results from these two models support Hypothesis 3.

**Table 3.** Model parameters for Hypothesis 3a in ovulatory cycles only (outcome variable is lnCRP)

Fixed effects						
					95% CI for β	
Parameter	β	***SE***	*t*	*P*	Lower	Upper
Intercept	−1.563	0.266	−5.881	<0.001	−2.094	−1.033
Age^a^	0.024	0.033	0.734	0.466	−0.042	0.090
BMI^a^	0.205	0.041	5.052	<0.001	0.123	0.287
SES						
Better-off	0.579	0.267	2.166	0.035	0.042	1.116
Poorer	(reference)					
Partnership * Phase						
Unpartnered, Early phase	0.179	0.179	0.603	0.548	−0.412	0.770
Unpartnered, Late phase	0.327	0.300	1.093	0.278	−0.269	0.924
Partnered, Early phase	−0.186	0.141	−1.323	0.187	−0.463	0.091
Partnered, Late phase	(reference)					
Partnership * Phase * (Day-wrt-ovulation)^2^						
Unpartnered, Early phase	−0.001	0.002	−0.322	0.747	−0.005	0.003
Unpartnered, Late phase	−0.006	0.004	−1.437	0.152	−0.013	0.002
Partnered, Early phase	0.006	0.002	2.808	0.005	0.002	0.010
Partnered, Late phase	0.005	0.004	1.283	0.200	−0.003	0.012
**Random effects**						
	**Variance**					
**Factor**	**Estimate**	***SE***				
Per-woman intercept	0.203	0.151				
**Repeated measures**						
	**Variance**					
**Factor**	**Estimate**	***SE***				
First-order autoregressive diagonal	0.955	0.149				
*ρ*	0.856	0.026				

^a^Centered.

**Table:** Table **4**. Model parameters for Hypothesis 3b, in anovulatory cycles only (outcome variable is lnCRP)

Fixed effects						
					95% CI for β	
Parameter	β	***SE***	*t*	*P*	Lower	Upper
Intercept	−1.675	0.375	−4.473	<0.001	−2.434	−0.917
Age^a^	0.021	0.064	0.336	0.740	−0.109	0.151
BMI^a^	0.215	0.086	2.517	0.018	0.040	0.391
SES						
Better-off	0.545	0.554	0.984	0.334	−0.591	1.682
Poorer	(reference)					
Partnership * Phase						
Unpartnered, Early phase	0.189	0.518	0.364	0.718	−0.860	1.237
Unpartnered, Late phase	0.026	0.546	0.047	0.962	−1.072	1.124
Partnered, Early phase	0.165	0.190	0.870	0.386	−0.210	0.540
Partnered, Late phase	(reference)					
Partnership * Phase * (Reverse day + 14)^2^						
Unpartnered, Early phase	0.009	0.003	3.331	0.001	0.003	0.014
Unpartnered, Late phase	−0.001	0.004	−0.142	0.887	−0.009	0.008
Partnered, Early phase	0.008	0.005	1.579	0.116	−0.002	0.017
Partnered, Late phase	0.002	0.004	0.430	0.668	−0.007	0.010
**Random effects**						
	**Variance**					
**Factor**	**Estimate**	***SE***				
Per-woman intercept	0.563	0.454				
**Repeated measures**						
	**Variance**					
**Factor**	**Estimate**	***SE***				
First-order autoregressive diagonal	1.217	0.470				
*ρ*	0.906	0.041				

^a^Centered.

Note that none of the changes in lnCRP occurring at the transition from follicular to luteal phase (i.e. at ovulation in ovulatory cycles) or between early and late phases (i.e. mid-cycle in anovulatory cycles) was significant.

In addition, a comparison of the models fitted to test hypotheses 1 and 3 (Figs 1–3) indicates that (for a given SES and having controlled for BMI and age) predicted CRP during early cycle was higher in anovulatory cycles (regardless of partnership status) than in ovulatory cycles from partnered women. Indeed, the highest concentrations of CRP during early cycle phase were associated with anovulatory cycles and the lowest CRP concentrations were associated with unpartnered ovulatory cycles.

### Clinical implications: CVD risk

**Figure 4.**
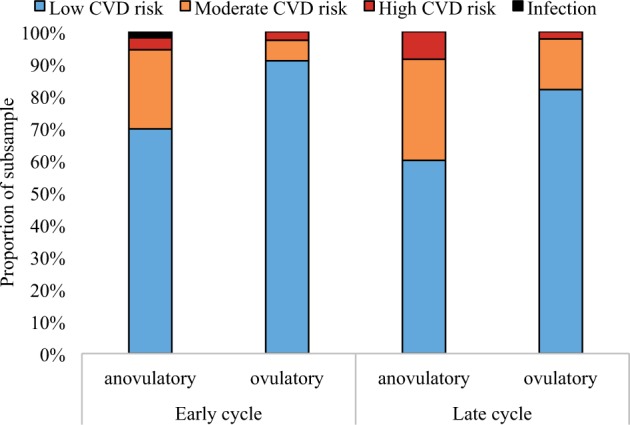
Cardiovascular (CVD) risk category (according to AHA guidelines) differs by cycle phase (early cycle: before Day 14; late cycle: after Day 14) and ovulation status

As CRP is used as a marker of risk for CVD, we investigated the clinical significance of the patterns of change in CRP reported above. We coded CRP values as low, moderate or high CVD risk according to the clinical interpretation guidelines recommended by the AHA [32]. We then tested whether there was a significant interaction between ovulation status and cycle phase (early-cycle, or before Day 14, vs late-cycle, or after Day 14) in predicting CVD risk category using a *χ^2^*-test for independence. There was a significant interaction between ovulation and cycle phase in predicting CVD risk category (*χ^2^*(3) = 16.20, *P* = 0.01), such that at early cycle, women with anovulatory cycles were significantly less likely to fall into the low risk category (*χ^2^*(3) = 15.44, *P* = 0.01). At late cycle, the difference between ovulatory and anovulatory cycles in CVD risk categories was marginally significant (*χ^2^*(2) = 5.62, *P* = 0.06, Fig. 4).

## CONCLUSIONS AND IMPLICATIONS

Inflammation is at the core of many critical and complex clinical problems, from treating chronic pain to diagnosing CVD risk to preventing obesity. Applying an evolutionary perspective in research on natural variations in inflammation, and trade-offs between immunity and other key processes such as reproduction, can open up new avenues for addressing these contemporary health challenges.

### Novel contributions to literature

Researchers as early as Darwin have posited the existence of trade-offs [24] (Darwin’s thoughts on the coordinated organization of an organism’s functions as noted in *On the Origin of Species*, p. 11–12: ‘Hence, if man goes on selecting, and thus augmenting, any peculiarity, he will almost certainly unconsciously modify other parts of the structure, owing to the mysterious laws of the correlation of growth’, and p. 178, ‘the whole organization is so tied together, during its growth and development, that when slight variations in any one part occur and are accumulated through natural selection, other parts become modified’.). However, the *mechanisms* behind such trade-offs still are being actively elucidated.

To the best of our knowledge, this is the first study to document the association of CRP profiles with ovulation status and sexual activity, and to do so in a population with higher pathogen load and less access to health care and other resources than are typically found in Western/industrialized nations. We observed evidence of trade-offs between immune status and reproductive functioning. Inflammation early, but not late, in the cycle was associated with anovulation. Ovulatory cycles were characterized by a CRP nadir in the days surrounding ovulation (i.e. mid-cycle); anovulatory cycles were not. High inflammation late in one cycle did not predict anovulation in the subsequent cycle, suggesting that inflammation-related ovarian suppression exhibits within-cycle specificity. Finally, we found significant interactions between cycle phase, sexual partnership and inflammation: specifically, in ovulatory cycles the observed mid-cycle decline in inflammation was seen in women investing in reproduction (i.e. regularly sexually active) but not in those who were not investing in reproduction (i.e. sexually abstinent).

Interestingly, among ovulatory cycles, early-phase inflammation in partnered women’s cycles was higher than that of unpartnered women (but still lower than that seen in the anovulatory cycles of either partnered or unpartnered women). Exposure to pathogens from the intimate partner [93], or vaginal irritation due to sexual activity and/or exposure to condoms [94], can prompt immune response including mild inflammation; however, responding with anovulation to this moderate rise in inflammation would prevent conception. The female reproductive system may attenuate responsiveness to inflammation under the conditions of partnership; that is, sexual activity and/or sexual partnership may tip the balance between reproduction and immunity in favor of reproduction. This may occur via alterations in hypothalamic receptor sensitivity for inflammatory cytokines [95], which would in turn blunt the signal received by the HPG (hypothalamic-pituitary–gonadal) axis. Broadly, these findings support the hypothesis that ovarian cycle variation in inflammation contributes to coordination of the trade-off between reproduction and immunity.

This Bolivian study population, alongside other studies in less-industrialized nations and populations experiencing significant environmental stress [54–57, 66, 67, 96, 97], offers a valuable counterpoint to the extant clinical literature. The vast majority of research on CRP and female reproductive health has been conducted in white women of middle to high SES living in wealthier (industrialized/post-industrialized) nations. Such limited representation of human variation is problematic given the evidence that women living in these wealthier societies differ significantly in diet, lifestyle, and reproductive history from those in poorer, less-industrialized and agrarian societies and consequently, differ significantly in the degree and chronicity of inflammation [98]. The participants in our study experienced significant physiologic stress from energetic demands (chronically low calorie and/or nutrient intake, and a lifestyle more physically demanding than typical of post-industrialized populations), a cold and arid climate, and limited financial assets. In addition, despite relatively higher exposure to pathogens, Bolivians typically have a much lower exposure to immunoactive medications (e.g. antibiotics) than individuals living in wealthier nations [99–101].

Although such differences between poorer and wealthier countries can be expected to have important consequences for immune function, it is noteworthy that even within this Bolivian sample, the poorest women exhibited significantly lower CRP than the subsample of better-off women. Similarly, in Ecuadorian lowland men and women, McDade *et al.* [63] found relatively lower CRP among individuals living in areas with high rates of infections compared with US populations under low risk of infection (but see also [57] for contrasting findings from a Bolivian lowland population). Such differences may have immunodevelopmental roots: for instance, Philippine adults who had experienced higher levels of microbial exposure in infancy [54] have lower levels of CRP. Although our data are valuable for being among the first to examine inflammation-related anovulation in a non-industrialized population, obviously they are by no means representative of all non-industrialized populations. Examining the diversity of the human species across habitats, climates and cultures will strengthen our models of immune response and reproduction [25, 38].

### Context within previous research

Published findings on variation in CRP across the ovarian cycle of healthy women in Western populations are mixed, with reports that CRP peaks at mid-cycle, nadirs at mid-cycle, rises across the cycle, or does not vary systematically across the cycle [74–78]. This wide variety of findings may be due in part to differences in inclusion and/or accounting for anovulatory cycles. One study that compared ovulatory and anovulatory cycles found differences in CRP at the early and mid-cycle time-points but not late cycle, with higher CRP in ovulatory than anovulatory cycles [76]. Another study that controlled for differences in luteal phase P4 in their sample of ovulatory and anovulatory cycles showed a U-shaped curve with lowest CRP around ovulation [78]. Taken together with the present study, these findings suggest that ovulatory and anovulatory cycles differ significantly in patterns of CRP, a finding also supported by studies of ovarian wave dynamics [43]. Of note, this may also explain why reports about the association of CRP with variations in ovarian hormones have been so inconsistent, with some reports suggesting that E2 or P4 is pro-inflammatory and others, anti-inflammatory [74, 77, 78]. It is possible that ovulation itself—a distinct phenomenon that is related to, but not entirely covariant with, hormone concentrations [81, 102]—is more pertinent to understanding changes in CRP during the ovarian cycle than are ovarian hormone concentrations *per se*. There is evidence of such direct crosstalk between immune and reproductive systems, with cytokines acting on neural circuits that mediate sexual behavior, and potentially, vice versa [103].

Other reports indicate that sexual partnership influences reproductive hormone profiles across the menstrual cycle in healthy women, such that sexually partnered women have lower testosterone but higher mid-cycle estradiol and luteal phase progesterone than unpartnered women [84, 104, 105]. These studies frequently have been limited by lack of consideration of ovulation or anovulation rates. However, given the work showing higher rates of ovulation among sexually partnered women [82–85], it is likely there is a significant role for the social and sexual environments as predictors of ovarian function, and, by extension, immune function.

Several studies have found significant differences between partnered and unpartnered women in terms of endocrine function [104, 106–109], and, among healthy individuals, immune function [49]. Compared with unpartnered women, partnered women have higher estradiol but lower testosterone, and higher luteal progesterone. Higher estradiol early in the cycle (that is, in the absence of progesterone) is associated with higher markers of inflammation such as interleukin-6 [110]; thus, partnered women are likely to have higher levels of early-cycle inflammation than unpartnered women.

Analyses reported in previous studies of CRP across the ovarian cycle routinely exclude data from participants with CRP levels over 3 mg/l, as the AHA and the Centers for Disease Control have defined CRP values >3 mg/l as ‘pathological’ and not reflecting healthy individuals. However, the ‘pathology’ in this case is CVD [29] and not frank infection (during which CRP levels are closer to 10–100 mg/l) [111]. Eliminating values over 3 mg/l restricts our understanding of CRP variation during the ovarian cycle by disregarding the normal function of inflammation, which is to increase dramatically but transiently in response to infection or wound healing. In wealthier populations, exposure to pathogens is typically lower and elevated CRP thus more typically reflects pathology; similarly, anovulation in wealthier populations is often associated with estrogen dysregulation and thus has been characterized as a pathological state [112].

However, our findings suggest that in populations exposed to environmental stressors demanding an immune response, anovulation may be adaptive. Several studies have suggested that high pathogen exposure early in life leads to higher adult immune response to pathogens, but also lower chronic inflammation in adulthood [25, 63]. This in turn may help balance reproductive and immune investments because inflammation is, for these populations, a signal of an acute state. In a large, well-conducted trial in healthy US women, Gaskins *et al.* [78] found that although 32% of women had elevated CRP values during at least one point of their cycle, CRP values were consistently high across multiple cycles in only 2% of cases; in other words, high inflammation in one measurement within one cycle rarely predicted high inflammation in other measurements. This pattern of CRP elevations as generally transient further reflects the dynamic nature of inflammation, and its responsiveness to changing conditions within the body.

### Clinical implications

The hypothesis that anovulation may be an adaptive, flexible strategy deployed to regulate reproductive fitness on a cycle-by-cycle basis vis-à-vis inflammation load suggests important clinical implications.

First, given CRP’s role as a marker of CVD and other inflammation-related conditions, variations in CRP across the ovarian cycle can confound clinical interpretation. Recent studies have suggested that failure to account for ovarian cycle variation in CRP contributes to a 3-fold over-diagnosis of CVD risk in healthy women [86]. We found that healthy women with anovulatory cycles were significantly more likely to be classified as moderate or high risk of CVD according to the AHA-recommended classification system if tested early in their cycle rather than late cycle [32]. That is, if a women presents for evaluation of her CVD risk during an anovulatory cycle, there is a significant chance she will be differently classified (and likely misclassified) if she presents early versus late in her cycle. These findings suggest that in women of reproductive age, single tests of CRP may have limited prognostic value, and instead support tracking patients’ CRP over time or, at a minimum, scheduling a test at about the end of the second week of the cycle, a time when our findings suggest that CRP in healthy women is most likely to be at a nadir in all cycles regardless of ovulation or partnership status.

Second, the chronicity of inflammation may have a greater impact on reproduction than does the degree. That is, because acute inflammation appeared to impact ovulation on a cycle-by-cycle basis, we would expect that ongoing ovarian disruption would be due to ongoing inflammation rather than high inflammation *per se*. Moreover, anti-inflammatory treatments (including diets and lifestyle changes) are increasingly recommended as first-line interventions for many diseases, including infertility and polycystic ovarian syndrome (PCOS). For example, Boots and Jungheim [113] argued that anti-inflammatory behavioral treatments may improve both pain and poor folliculogenesis in women with PCOS. Our findings suggest that such treatments would have the greatest impact on ovulation if administered early in the current ovarian cycle. Indeed, anti-inflammatory treatments administered in mid- or late-cycle may have little effect on anovulation-related infertility.

### Strengths and limitations

Limitations of our study include a moderate sample of participants (*n* = 61) (however, sample sizes in all but one published study [78] were *n* ≤ 36). Because hormone data were only available for every other day, our maximum precision for determining timing of ovulation was a range of 2 days.

As CRP is a non-specific marker of inflammation, the precise cause of high CRP (infection, wound healing, diet, stress, or some other factor) cannot be ascertained. Similarly, we cannot know, based on this study alone, which of the many aspects of a sexual partnership underlie the associations between reproduction and immune function reported here. Future studies should further examine the mechanisms that drive the observed interactions between partnership, hormones and inflammation.

It is also worth noting that selection bias for less fecund women can occur in a cross-sectional study of menstruating women in a natural fertility population because the most fecund women are likely to be either pregnant or lactating, and hence unavailable for such a study [38, 114]. Although our sample has demonstrated high fertility (median parity to date was three births for the currently partnered women, with each birth followed by a prolonged period of lactation, during ∼8 years of partnership) [52, 81], it is possible that our sample in this study is biased in this manner and that the relationships among inflammation, ovulation and partner status that we observed differ in some way between women of higher and lower fecundity. To evaluate this possibility requires additional (e.g. longitudinal) research.

Whether our results apply to women outside the age range of our sample also remains to be tested. This was a sample of young women who were relatively early in their childbearing years. Life-history theory suggests that the threshold to reproductive investment is lower in older women (particularly those without prior offspring) [25]. Thus, potentially, anovulation would not be associated with inflammation in an older sample or would be associated with higher inflammation levels than were observed in our sample.

Strengths of our study include multiple consecutive cycles for each woman, multiple CRP measurements during the 2 weeks around mid-cycle, determination of ovulation/anovulation based on well-characterized hormonal profiles, good statistical controls for individual differences in baseline CRP and a novel population.

### Conclusions

We identified a link between markers of inflammation and ovarian function, whereby ovulation was associated with a mid-cycle decrease in inflammation while anovulation was associated with high inflammation early in the ovarian cycle. We also found evidence for cycle-by-cycle specificity of inflammation patterns, such that inflammation in one cycle did not influence ovulation in other cycles (and vice versa). This study also demonstrates a need to consider social factors such as sexual behaviors as powerful signals that help coordinate immune activity, which in turn may regulate reproductive investment via permitting or disrupting ovulation. Although further work is needed to elucidate the mechanisms underlying these effects, our findings suggest an important role for sociosexual behavior in balancing reproductive and immune priorities. Harnessing such effects may prove to be transformative in preventing sexually transmitted diseases and promoting women’s sexual health. Moreover, the findings speak directly to the clinical interpretation of CRP as a biomarker of CVD risk, as there is significant variation in CRP (and thus, the apparent associated risk prognosis) within the same (healthy) individual during the course of the ovarian cycle, and with partnership status in ovulatory cycles. The translational import of our findings suggests that clinical interpretation of inflammation biomarkers such as CRP without including consideration of reproductive variables (ovarian cycle phase, sexual activity status) is at best incomplete and at worst inaccurate. This is the first such study in a non-industrialized population, and contributes to a growing literature on life history and the ecology of women’s immune function. As such, this and future studies will provide a foundation for understanding how trade-offs between reproduction and immunity play out in women worldwide.

## Supplementary Material

Supplementary Data
