# Supplementary material for: Links among inflammation, sexual activity and ovulation: Evolutionary trade-offs and clinical implications
Source: Evol Med Public Health. 2015 Dec 16;2015(1):304–24. doi: 10.1093/emph/eov029 (PMC4681377; doi:10.1093/emph/eov029)
Supplement: Supplementary Data [file supp_2015_1_304__index.html]

Links among inflammation, sexual activity and ovulation — Supplementary Data 

# Links among inflammation, sexual activity and ovulation

## Supplementary Data

files

- Supplementary Data - pdf file
