# Supplementary material for: Links among inflammation, sexual activity and ovulation: Evolutionary trade-offs and clinical implications
Source: Evol Med Public Health. 2015 Dec 16;2015(1):304–24. doi: 10.1093/emph/eov029 (PMC4681377; doi:10.1093/emph/eov029)
Supplement: Supplementary Data [file supp_eov029_Supplementary_Appendix.pdf]

C-reactive protein high-sensitivity europium-labeled biotin-strepavidin assay system details

Summary: 96-well microplate was coated with streptavidin A (a high-affinity binding molecule). A CRP-specific capture antibody was treated to attach biotin (the streptavidin binding molecule) and europium (a phosphorescent signal). To reconstitute samples, a 1/8'' section of sample papers was punched from the dried blood spots and transferred to tubes with DELFIA assay buffer. The resulting sample solutions were pipetted into the streptavidin-prepared microplate with the biotinylated europium-labeled CRP antibodies; plates were allowed to incubate, then washed with assay buffer. Fluorescence of the remaining (i.e., bound) europium was read in a fluorometer; these values were directly proportional to CRP concentrations in each well.

Details: Streptavidin A (Invitrogen #43-4302) was added to 100 mM citric phosphate buffer (pH 5.0) remaining overnight at room temperature. 100 μl of the Streptavidin A working solution were added to each well for a final concentration of 0.5 μg/well.

To biotinylate CRP monoclonal antibodies, first, sodium azide was removed from anti-CRP monoclonal antibodies (clone C2) using PD-10 equilibrated in PBS (pH 7.4) at 4°C. The purified antibodies were concentrated to 4 mg/ml and labeled with Biotin (Biotin-XX-SSE) for one hour at room temperature and then shook at 400 rpm. These biotinylated anti-CRP monoclonal antibodies were purified by desalting on a PD-10 column and protein content of each fraction was monitored via A280. Fractions with concentrations below 0.1 mg/ml were pooled. After that, labeled antibodies were filtered using a .22 μm syringe filter and biotin was quantified using Pierce's HABA Biotin Quanitation Kit. An average of 7 moles of Biotin per mole of IgG were incorporated.

CRP monoclonal antibodies were also labeled with europium. Anti-CRP monoclonal antibodies (clone C6) were dialyzed overnight using 1 liter of 100 mM Carbonate Buffer (pH

9.3) at 4°C. The concentration of dialyzed antibodies was adjusted to 2 mg/ml. For labeling to occur, 120 molar excess of anti-CRP monoclonal antibodies, clone C6, were reacted with 1200 mmol/L Eu-N^1-ITC chelate in a borosilicate glass test tube for 20-24 hours at 4° C and shook at 400 rpm.

Excess chelate was removed by diluting the reaction in 4 ml of TSA 50 mM Tris-HCl, 0.9% NaCl, and 0.1% NaN_3 at pH 7.8) and concentrated to 500 µl using a centrifugal filter (Amicon). Labeled antibodies were further purified on a PD-10 equilibrated in TSA and collected in 250 µl fractions with protein content estimated via A280. An average of 6 moles of europium per mole of IgG were incorporated.

Streptavidin A was coated on a mirotitre plate and bound the biotinylated capture antibody to CRP clone, C2. A second antibody labeled with europium, then bound to the Streptavidin A Biotin – C2 – CRP complex. Using Delfia enhancement solution, europium was removed and amount of fluorescence was directly proportional to the CRP concentration in each well.

After reagents necessary to carry out the assay were prepared, using a hole punch, one 1/8” spot was punched out from each sample spot. Using tweezers, discs were then transferred to appropriate tubes. 125 µl of the Delfia assay buffer (pH 7.75) were then added to the tube. Tubes were vortexed and rotated on an automatic shaker at 300 rpm for 1.5 hours at room temperature. Next, the Streptavidin coated mirotitre plate was washed two times with 350 µl of the Delfia assay buffer. Paper towel was used to blot the plate to ensure removal of liquid from the wells. To block the plate, 200 µl of the Delfia assay buffer were added to the plate and the plate shook at 400 rpm for 1.5 hours, rotated 180° after 45 minutes. The blocked plate was then washed 3 times using 350 µl of the Delfia wash buffer and wells were blotted with a paper towel. 50 µl of

the eluate were pippeted into appropriate wells. Then, the reactant working solution was made by the addition of 12 mL of Delfia assay buffer to C2-Biotin and C6-Europium, both of which were added resulting in a final concentration of 0.5 ng/µl each. This solution was vortexed and 100 µl of it were transferred to each well and left to incubate at room temperature on the automatic shaker at 400 rpm for 2 hours, and the plate was rotated 180° after 1 hour. After incubation, plates were washed 8 times with 350 µl of Delfia wash buffer and 200 µl of Delfia enhancement solution (pH 3.2) were then added to each well. Finally, samples were run through a flourometer to detect concentrations of CRP.

The mean values of the CRP quality controls were 0.022, 0.259, 1.208, and 3.271 mg/L for the low, medium, high, and very high controls, respectively. The interassay CVs were 14.4%, 14.9%, 12.3% and 10.9% while the intrassay CVs were 2.0%, 1.2%, 1.6%, and 1.4%, respectively. The MDD for the CRP assay was 0.030 mg/L. CRP monoclonal antibodies did not cross-react with serums from dog, cat, horse, mouse, or rat.

Hormone assay performance characteristics

Assays for E2 and P4 used kit reagents from Diagnostic Systems Laboratories (E2, DSL-4800; P4, DSL-3400). Assay performance characteristics include MDD 4.2 pg/mL and 0.17 ng/mL for E2 and P4 respectively; intraassay and interassay CVs for E2 of 19.8%, 15.2% and 9.2%, and of 34.8%, 15.6%, and 29.7%, respectively, on low, medium, and high controls (mean 13.6, 38.5, and 129.0 pg/mL), and for P4 of 11.7%, 7.607%, and 2.5%, and of 29.0%, 17.1%, and 6.1%, respectively, on low, medium, and high controls (mean 0.61, 4.47, and 12.86 ng/mL). Gonadotropin assay performance characteristics include MDD 0.13 U/L and 0.26 for FSH and LH respectively; intraassay and interassay CVs for FSH of 5.3%, 7.4% and 9.1%, and of 7.8%,

9.9% and 9.0%, respectively, on low, medium, and high controls (mean 3.89, 7.17, and 18.01 U/L), and for LH of 10.9%, 3.6%, and 11.3%, and of 13.5%, 20.8% and 19.0%, respectively, on low, medium, and high pools (mean 0.98, 9.72, and 32.54 U/L).).
